# Circulating Levels of Remnant Cholesterol as Potential Biomarkers for Predicting Gestational Diabetes: A Systematic Review

**DOI:** 10.1155/jdr/9942007

**Published:** 2026-09-07

**Authors:** Fatemeh Abdi, Fatemeh Alsadat Rahnemaei, Fatemeh Khosravani, Farzad Sadri, Yaser Mohammadi

**Affiliations:** ^1^ Nursing and Midwifery Care Research Center, Health Management Research Institute, Iran University of Medical Sciences, Tehran, Iran, iums.ac.ir; ^2^ Student Scientific Research Center (SSRC), Tehran University of Medical Sciences, Tehran, Iran, tums.ac.ir; ^3^ Student Research Committee, Shahid Sadoughi University of Medical Sciences, Yazd, Iran, ssu.ac.ir; ^4^ Geriatric Health Research Center, Birjand University of Medical Sciences, Birjand, Iran, bums.ac.ir; ^5^ Student Research Committee, Iran University of Medical Sciences, Tehran, Iran, iums.ac.ir; ^6^ Department of Biochemistry, Faculty of Medicine, Iran University of Medical Sciences, Tehran, Iran, iums.ac.ir

**Keywords:** biomarkers, gestational diabetes mellitus, pregnancy, remnant cholesterol

## Abstract

**Objectives:**

Gestational diabetes mellitus (GDM) is a common pregnancy complication with serious consequences for both mother and child. Current diagnostic methods are invasive and limited. The present study was conducted to evaluate circulating remnant cholesterol (RC) as a potential predictive and diagnostic biomarker for GDM.

**Methods:**

The present study was conducted following PRISMA guidelines. Databases including Scopus, PubMed, Web of Science (WoS), Embase, ProQuest, Cochrane, and Google Scholar were searched up to August 2025 using standardized keywords. The search results were independently screened by two researchers in two stages. The quality of included articles was assessed using the Newcastle–Ottawa Scale (NOS).

**Results:**

Finally, 11 studies were included in this systematic review. Women who developed GDM had higher circulating RC levels than those who did not. Overall, higher RC levels in early pregnancy were linked to a greater risk of GDM, with even small increases in RC translating into a higher likelihood of the condition. Although the strength of this link varied across studies and one suggested a possible protective effect, the evidence points to a fairly consistent association.

**Conclusions:**

This systematic review indicates that circulating RC is a promising candidate biomarker for early risk stratification of GDM. RC levels in the first trimester show a significant positive association with GDM risk, highlighting its potential for clinical integration. Further studies are needed to define cutoffs, validate across populations, and confirm real‐world applicability.

## 1. Introduction

Gestational diabetes mellitus (GDM) affects approximately 6%–15% of pregnancies worldwide and is associated with significant short‐ and long‐term complications for both the mother and the child [1]. This condition not only increases the risk of pregnancy and delivery complications such as preeclampsia, fetal macrosomia, and preterm birth, but also predisposes affected mothers to a higher risk of developing Type 2 diabetes, cardiovascular disease, and metabolic syndrome in the following decades [2]. Furthermore, children born to mothers with GDM are at an increased risk of developing obesity, diabetes, and metabolic disorders in adulthood [3]. An accurate and timely diagnosis of GDM is therefore crucial for preventing these complications; however, current diagnostic methods have significant limitations. The oral glucose tolerance test (OGTT), considered the gold standard for diagnosing GDM, is not only invasive and requires prolonged fasting, but also causes considerable discomfort, involves high costs, and necessitates multiple blood samples [4]. These limitations, along with the increasing need for earlier detection of GDM, have driven researchers to search for new and noninvasive biomarkers that can offer high sensitivity and specificity, as well as strong predictive power for diagnosing this condition [5].

Among potential biomarkers, the lipid profile, particularly various cholesterol fractions, has received considerable attention. Remnant cholesterol (RC), which constitutes a portion of cholesterol carried by triglyceride‐rich lipoproteins including VLDL, IDL, and chylomicrons, has emerged as a promising biomarker [6]. This fraction, calculated using the formula RC = TC − HDL − C − LDL − C, plays a key role in lipid metabolism and, due to its atherogenic and proinflammatory effects, is recognized as an independent risk factor for cardiovascular diseases [7].

The importance of RC in the pathophysiology of metabolic disorders, particularly insulin resistance, has become increasingly evident in recent years [8, 9]. Extensive research indicates that elevated levels of RC are not only associated with a higher risk of cardiovascular disease and related mortality but also serve as a strong predictor for the development of Type 2 diabetes and metabolic syndrome [10]. The biological mechanisms through which RC exerts its effects include the promotion of systemic inflammation, accumulation within vascular walls, interference with insulin signaling, and induction of oxidative stress—all of which contribute to the pathogenesis of metabolic disorders [11]. Pregnancy is accompanied by profound physiological changes in lipid metabolism, aimed at meeting maternal energy demands and ensuring optimal fetal growth. These changes include significant increases in cholesterol and triglyceride synthesis, as well as alterations in lipoprotein composition and function [12]. Under normal conditions, these changes occur gradually and in alignment with the different stages of pregnancy. However, in women predisposed to GDM, these processes may become dysregulated and pathological. Studies have shown that women with GDM exhibit higher levels of triglycerides, total cholesterol, VLDL, and unfavorable lipid ratios, which may reflect underlying disturbances in RC metabolism [13].

Preliminary scientific evidence suggests a potential role of RC in the development of GDM. Findings from Wang et al. indicated that RC levels are elevated in women with GDM compared with healthy controls [14]. Similarly, Li et al. demonstrated that higher RC concentrations are associated with an increased risk of GDM [15]. Consistent with these findings, Cheng et al. reported that elevated RC levels in early pregnancy are associated with a higher likelihood of developing GDM [16]. However, results across studies have been somewhat inconsistent and occasionally contradictory. Therefore, the present systematic review was conducted to comprehensively evaluate the existing evidence on circulating RC levels as a potential biomarker for the prediction and diagnosis of GDM.

## 2. Methods

### 2.1. Design and Registration

The study protocol was prospectively registered in PROSPERO (International Prospective Register of Systematic Reviews), assigned the unique identifier code CRD420251116615. This systematic review adhered to the methodological guidelines outlined in the PRISMA (Preferred Reporting Items for Systematic Reviews and Meta‐Analyses) framework (Table S1) [17].

### 2.2. Search Strategy

A comprehensive literature search was conducted across multiple electronic databases—Web of Science (WoS), PubMed/MEDLINE, Embase, Proquest, Scopus, and Google Scholar—with an inclusion cutoff date of 6th August 2025. To ensure thorough coverage, manual searches were performed on the websites of key organizations (e.g., http://who.org) and within the reference lists of eligible studies. The search strategy involved an iterative process: initial searches employed individual keywords, which were subsequently combined using Boolean operators (“AND”/“OR”) to generate refined search phrases. The following search string was applied across all databases to retrieve relevant studies: (“Gestational Diabetes” OR “Gestational Diabetes Mellitus” OR GDM OR “Pregnancy‐related diabetes”) AND (“remnant cholesterol” OR “remnant lipoproteins” OR “remnant‐like particle cholesterol” OR “remnant lipoprotein cholesterol” OR “RC levels”). A manual search for references from eligible articles as well as from published review articles was also employed to identify additional relevant studies.

### 2.3. Eligibility Criteria

PECOS inclusion criteria are as follows:

• Population (P): pregnant women diagnosed with GDM.
• Exposure (E): RC.
• Comparison (C): healthy pregnant women (without GDM).
• Outcome (O): incidence or association with GDM.
• Study designs (S): cohort and case‐control studies.

Exclusion criteria are as follows:

• Letters.
• Commentaries.
• Short communications.
• Conference abstracts.
• Grey literature.
• Review articles.
• Any studies deemed irrelevant to the analysis were excluded.

### 2.4. Study Selection

**Figure 1:**
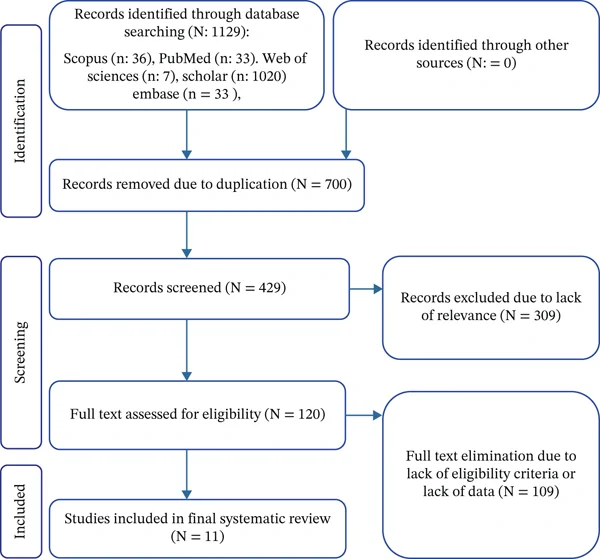
The literature search results and the screening process based on the PRISMA flowchart.

The search results were imported into EndNote 21. After removing duplicates, titles and abstracts were independently screened by two independent reviewers (F.A., F.A.R.) for relevance. Full‐text articles of potentially eligible studies were then assessed based on predefined inclusion and exclusion criteria. For studies with unclear or insufficient information, the corresponding authors were contacted for clarification. Any discrepancies between reviewers were resolved through discussion or consultation with a third reviewer (Y.M.) (see Figure 1).

### 2.5. Data Extraction

The eligible studies were independently reviewed by the two authors (F.K., F.S.), and then assessed their quality was assessed. Subsequently, they reexamined their perspectives and resolved any existing disagreements. In Table 1, a comprehensive summary of the extracted information is presented. For each included study, we systematically extracted comprehensive data to ensure robust analysis. General study characteristics included the first author′s name, publication year, study design, and geographic region. Participant demographics encompassed sample size, mean age with standard deviation, body mass index (BMI), gestational age in weeks, and gravidity. Regarding RC assessment, we documented the measurement method (direct or calculated), reported levels with units (mg/dL or mmol/L), and timing of evaluation (e.g., second trimester). The diagnostic criteria for GDM were recorded, including specific methods like OGTT, IADPSG, or WHO standards. For effect measures, we extracted odds ratios (ORs) or relative risks (RRs) along with 95% confidence intervals (CIs), as well as any adjusted covariates included in the analysis models (e.g., age, BMI, family history). Finally, we summarized key findings from each study. This standardized data extraction protocol enabled rigorous quantitative synthesis and qualitative evidence evaluation while maintaining methodological consistency across all included studies.

**Table 1:** Contextual details of included studies in the systematic review.

ID	Author (ref)	Year	Design	Region	Sample size (*n*)	Age (year)	BMI (kg/m2)	GA (weeks)	Gravidity	RC measure (mmol/L)	Evaluation time	GDM diagnosis	Effect size (OR:/RR: (CI))	Adjusted variables	Key points
1	Su et al. [19]	2024	Cohort	China	T: 33018	T: 31.96 ± 3.83	T: 24.0–27.9	6 to 13	Nullipara: 17689	Calculation method	First trimester	OGTT	OR: 2.254 (1.943–2.615)	Age, BMI, FBG, number, gravidity, Education level, employment status, family income, smoking, alcohol consumption.	• Positive and significant association between increased RC and a higher risk of GDM.
					GDM: 5017	GDM: 32.55 ± 4.00			Multipara: 15329	GDM: 0.73 ± 0.17					
					Non‐GDM: 28001	Non‐GDM: 31.75 ± 3.67				Non‐GDM: 0.4 ± 0.2					
2	Gue et al. [20]	2024	Cohort	China	T:830	T: 28.5 ± 4.0	T: 21.0 ± 2.6	4 to 8	Not reported	Calculation method	First trimester	OGTT	OR: 1.09 (1.00–1.14)	Age, BMI, fertilization method, TC, HDL‐C, hepatic steatosis, complement components (C3, C4, C1q), D‐dimer, coagulation factors.	• With every 0.1 unit increase in RC, the risk of developing GDM increases by
					GDM: 121	GDM: 29.7 ± 4.0	GDM: 20.9 ± 2.6			GDM: 0.4 ± 0.22					
					Non‐GDM: 709	Non‐GDM: 28.3 ± 4.0	Non‐GDM: 21.6 ± 2.7			Non‐GDM: 0.4 ± 0.14					9%, and this association is statistically significant.
3	Shan et al. [9]	2025	Cross‐sectional	United States	T:1590	GDM: 30.96 ± 7.22	GDM: 30.96 ± 7.22	Not reported	Not reported	NHANES	First trimester	FPG	OR: 2.6 (2.0–3.3)	Age, BMI, gravidity, coagulation factors, gestational weight gain, ethnic origin, waist circumference, employment status, family income, smoking, fertilization method, and TC.	• An increase in the NHHR ratio (or RC) was significantly associated with a higher risk of developing GDM.
					GDM:297	Non‐GDM: 27.06 ± 5.63	Non‐GDM: 27.1 ± 5.63			GDM: 3.87 ± 1					
					Non‐GDM: 1293					Non‐GDM: 3.47 ± 1.16					
4	Li et al. [15]	2025	Cohort	China	T:1361	T: 29 ± 3	T: 21.48	10 to 14	Not reported	Calculation method	First trimester	OGTT	OR: 1.05 (1.02–1.08)	Age, BMI, FBG, hemoglobin, alcohol consumption, fertilization method, HDL‐C, LDL‐C, AST, and HDP.	• Positive and significant association between increased RC and a higher risk of GDM.
					GDM:353	GDM: 30	GDM: 22.7			GDM: 0.63					
					Non‐GDM: 1008	Non‐GDM: 29	Non‐GDM: 20.82			Non‐GDM: 0.25					
5	Sheng et al. [21]	2025	Cohort	South Korea	T:582	T: 32.07 ± 3.78	T: 22.03 ± 3.49	10 to 14	Nullipara: 304	Calculation method	First trimester	OGTT	OR: 1.1 (1.065–1.136)	Age, BMI, insulin, HOMA‐IR, AST, ALT, and GGT.	• Positive and significant association between increased RC and a higher risk of GDM.
					GDM:35	GDM: 32.17 ± 4.14	GDM: 22.87 3.76		Multipara: 287	GDM: 0.85					
					Non‐GDM: 547	Non‐GDM: 31.56 ± 3.57	Non‐GDM: 21.24 ± 2.98			Non‐GDM: 0.42					
6	Sormunen‐Harju et al. [22]	2023	Cohort	Finland	T: 755	GDM: 34.0 ± 4.5	GDM: 35.8 ± 4.3	13 to 28	Nullipara:	NMR‐based metabolomics platform	First, second, and third trimesters	OGTT		Age, BMI, gravidity, family income, smoking, HDP, family history of diabetes, marital status, hormonal and inflammatory factors.	• The level of RC was positively and significantly associated with the GDM group only during the first trimester.
					GDM: 185	Non‐GDM: 32.6 ± 5.3	Non‐GDM: 34.3 ± 3.8		GDM: 37	GDM: 0.4 ± 0.3					
					Non‐GDM: 570				Non‐GDM: 209	Non‐GDM: 0.35 ± 0.25					
									Multipara;						
									GDM: 149						
									Non‐GDM: 360						
7	Gao et al. [23]	2023	Cohort	South Korea	T: 590	GDM: 32.54 ± 3.56	GDM: 25.95 ± 5.17	Up to 14	Nullipara:	Calculation method	First trimester	OGTT	OR: 1.458 (1.221–1.741)	Age, BMI, FBG, TG, HOMA‐IR, adiponectin, AST, ALT, and GGT.	• Positive and significant association between increased RC and a higher risk of GDM.
					GDM: 37	Non‐GDM: 32.03 ± 3.81	Non‐GDM: 21.78 ± 3.20		GDM:18	GDM: 0.804 ± 0.96					
					Non‐GDM: 553				Non‐GDM: 262	Non‐GDM: 0.595 ± 0.216					
									Multipara:						
									GDM: 19						
									Non‐GDM: 291						
8	Guo et al. [8]	2025	Cohort	China	GDM: 399	GDM: 29.12 ± 2.61	GDM: 21.07 ± 2.34	24 to 28	Not reported	Calculation method	Second trimester	OGTT	OR: 1.633 (1.079–2.588)	Age, BMI, FBG, 1 h‐PG, 2 h‐PG, TC, HDL‐C, and LDL‐C.	• The RC was significantly positively associated with the risk of adverse pregnancy outcomes (APOs) in women with GDM.
										GDM: 0.565 ± 0.61					
9	Cheng et al. [16]	2025	Cohort	China	T: 6619	T: 31 ± 3.7	T: 20.89 ± 2.69	Up to 13	Nullipara:	Calculation method	First or second trimester	OGTT	OR: 1.39 (1.18–1.64)	Age, BMI, FBG, HDP, Alcohol consumption.	• The RC has a moderate but significant association with an increased risk of GDM.
					GDM: 2054	GDM: 32 ± 3.7	GDM: 21.77 ± 3.07	14 to 27	T: 4426	GDM: 0.33 ± 0.22					
					Non‐GDM: 3565	Non‐GDM: 30 ± 3.7	Non‐GDM: 20.55 ± 2.41		GDM: 1307	Non‐GDM: 0.29 ± 0.215					
									Non‐GDM: 3119						
									Multipara:						
									T: 2193						
									GDM: 747						
									Non‐GDM: 1446						
10	Wang et al. [14]	2023	Cohort	China	T: 2528	T: 28.1 ± 3.4	T: 20.9 ± 2.8	Up to 16	Nullipara: 2158	Calculation method	Second trimester	OGTT	RR: 1.44 (1.09–2.31)	Age, BMI, gravidity, employment status, family income, smoking, LDL‐C, HDP, hormonal and inflammatory factors.	• Positive association between increased RC levels and the risk of diabetes.
					GDM: 256	GDM: 29.6 ± 3.7	GDM: 22.2 ± 3.4		Multipara: 370	GDM: 0.61 ± 0.49					
					Non‐GDM: 2272	Non GDM: 28.0 ± 3.3	Non‐GDM: 20.7 ± 2.7			Non‐GDM: 0.44 ± 0.34					
11	Lin et al. [24]	2025	Cohort	China	T: 13446	GDM: 30.4 ± 4.4	GDM: 21.9	Up to 13	Nullipara: 6302	Calculation method	First trimester	OGTT	RR: 1.5 (1.35–1.71)	Age, BMI, gravidity, BP, HDP, education level, cohort differences, lifestyle factors.	• Each unit increase in RC level, as well as each one standard deviation (SD) increase, was associated with a 124% and 114% increase in the risk of gestational diabetes mellitus, respectively.
					GDM: 2994	Non‐GDM: 30.4 ± 5.1	Non‐GDM: 21.15		Multipara: 3929	GDM: 1.52 ± 0.27					
					Non‐GDM: 10452					Non‐GDM: 1.25 ± 0.23					

Abbreviations: 1 h‐PG, 1‐hour postprandial glucose; 2 h‐PG: 2‐hour postprandial glucose; ALT, alanine aminotransferase; APO, adverse pregnancy outcomes; AST, aspartate aminotransferase; BMI, body mass index; CI, confidence interval; FBG, fasting blood glucose; GA, gestational age; GDM, gestational diabetes mellitus; GGT, gamma‐glutamyl transferase; HDL‐C, high‐density lipoprotein cholesterol; HDP, hypertensive disorders of pregnancy; HOMA‐IR, homeostatic model assessment of insulin resistance; ID, identifier; LDL‐C, low‐density lipoprotein cholesterol; NHANES, National Health and Nutrition Examination Survey; NMR, nuclear magnetic resonance; Non‐GDM, nongestational diabetes mellitus; NPO, normal pregnancy outcomes; OGTT, oral glucose tolerance test; OR, odds ratio; RC, remnant cholesterol; ref, reference; RR, relative risk; T, total; TC, total cholesterol.

### 2.6. Quality Assessment

The methodological quality of included cohort studies was evaluated using the NOS, a validated tool with established reliability [18]. This scale appraises studies across three key domains: (1) selection of study groups, (2) comparability of cohorts, and (3) assessment of outcomes. Each study could receive a maximum of 10 stars based on these criteria. We categorized study quality as follows: very good (7–9 stars), good (5–6 stars), satisfactory (4 stars), and unsatisfactory (0–3 stars). This standardized approach allowed for systematic quality assessment while maintaining consistency with established methodological practices in systematic reviews.

## 3. Results

### 3.1. Search Outcomes

Our initial search of the Scopus (n: 36), PubMed (n: 33), WoS (n: 7), Embase (n: 33), and Google Scholar (n: 1020) databases yielded a total of 1129 articles. After removing duplicates and reviewing titles and abstracts, we evaluated 120 full‐text articles for eligibility. Of these, 109 articles were excluded for not meeting inclusion criteria. This left us with 11 articles for review (Figure 1).

### 3.2. Studies Characteristics

The systematic review of the included studies revealed that most investigations were conducted in recent years (2023–2025), predominantly with a cohort design in Asian countries, particularly China (*n* = 7) and South Korea (*n* = 2), although studies from the United States and Finland (*n* = 1 each) were also reported. The sample sizes varied widely across studies, ranging from a few hundred participants to more than 33,000. The mean age of pregnant women in most studies was approximately 28–32 years, and their mean BMI was reported between 20 and 27 kg/m^2^. Most studies assessed RC levels during the first trimester of pregnancy (*n* = 7), although some studies extended the evaluation to the second trimester or even multiple stages of pregnancy. The diagnosis of GDM in almost all studies was based on the OGTT (*n* = 10), whereas a few studies employed alternative markers such as fasting plasma glucose or metabolomics (*n* = 1). Table 1 summarizes the characteristics of the included studies.

### 3.3. Quality Assessment Results

The quality assessment of the included studies based on the Newcastle–Ottawa Scale (NOS) indicated that the overall quality of the articles ranged from adequate to very high. As shown in Table 2, the total scores of the studies varied between 8 and 10 stars. Four studies achieved the maximum score of 10 stars, reflecting excellent methodological quality in terms of sample selection, group comparability, and outcome assessment. In addition, four studies received nine stars, whereas another three studies were rated with eight stars, indicating a moderate‐to‐high level of methodological quality.

**Table 2:** Quality assessment of the included studies by the “Newcastle–Ottawa” scale.

Author (year)	Selection (max 4★)	Comparability (max 3★)	Outcome (max 3★)	Total stars (max 10)
Cheng et al. (2025)	★★★★	★★	★★	8
Guo et al. (2025)	★★★	★★	★★★	8
Lin et al. (2025)	★★★★	★★★	★★	9
Li et al. (2025)	★★★★	★★	★★★	9
Sheng et al. (2025)	★★★	★★★	★★★	9
Shan et al. (2025)	★★★★	★★★	★★★	10
Gue et al. (2024)	★★★★	★★	★★	8
Su et al. (2024)	★★★★	★★★	★★★	10
Gao et al. (2023)	★★★★	★★★	★★★	10
Sormunen‐Harju et al. (2023)	★★★★	★★★	★★	9
Wang et al. (2023)	★★★★	★★★	★★★	10

### 3.4. Adjusted Variables Across Studies

Most articles accounted for a wide range of confounding variables in their adjusted analyses, including age, BMI, fasting blood glucose, socioeconomic status, reproductive history, inflammatory, and hepatic factors, as well as the method of fertilization. Table 1 reports the characteristics of the included articles. Across the 11 studies, age and BMI were consistently adjusted for, underscoring their central role as confounders in gestational diabetes risk. Nine studies also controlled for FBG, whereas five took gravidity into account. Lifestyle factors such as smoking and alcohol use were considered in six studies—mostly those conducted in China [9, 15, 16, 19–21]. Biological markers, including HDL‐C, LDL‐C, and HOMA‐IR, were included in five studies [8, 14, 15, 22, 23]. Notably, South Korean research [22, 23] placed particular emphasis on insulin resistance indicators, such as HOMA‐IR and adiponectin. A smaller number of studies [14, 20] adjusted for hormonal and inflammatory factors, highlighting some variability in how confounders were handled across the evidence base.

### 3.5. RC Levels and GDM Risk

**Figure 2:**
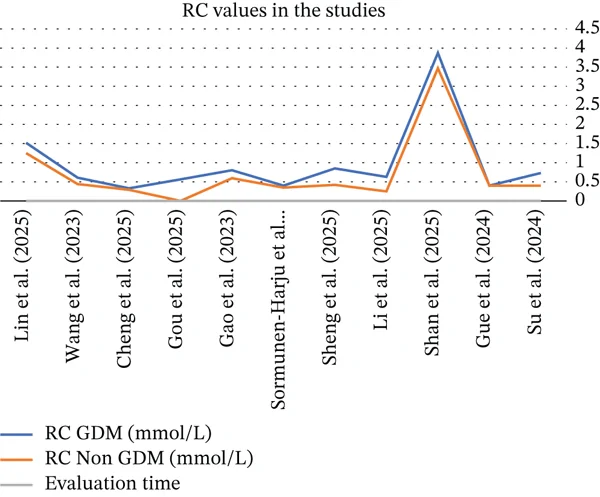
RC values across 11 studies between two groups.

**Figure 3:**
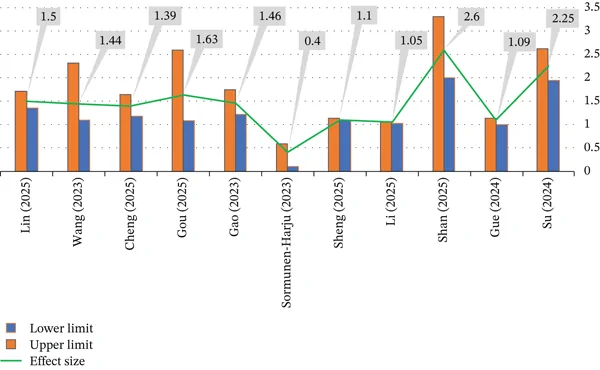
Effect size in each trimester of pregnancy across 11 studies.

Approximately, RC levels were higher in the GDM (0.97) compared with the non‐GDM group (0.71) (Figure 2). The findings indicated that elevated RC concentrations in the first trimester of pregnancy are positively associated with the risk of GDM. Specifically, each 0.1‐unit rise in circulating RC was linked to a 9% increase in the likelihood of developing GDM. However, another study showed that a one‐unit and one‐SD increase in RC levels was associated with a 124% and 114% increased risk of GDM after adjusting for potential confounding factors. The effect size of each study in each trimester of pregnancy was shown in Figure 3. According to this figure, most of the studies pointed in the same direction, showing that higher biomarker levels were generally linked to an increased risk of the outcome. The associations were particularly strong in Shan et al. (2024; OR = 2.6) [9] and Su et al. (OR = 2.254) [19], whereas Li et al. (OR = 1.05) [15] reported only a minimal increase in risk. Interestingly, Sormunen‐Harju et al. stood out as the only study suggesting a protective effect in nonobese GDM women in early pregnancy, with an OR of 0.4 [20]. Precision also varied across the studies: some, such as Gou et al. [24], Li et al. [15], Sheng et al. [22] had relatively narrow CIs, whereas others, like Su et al. (2024), showed much wider ranges, leaving more room for uncertainty. Altogether, the pattern that emerges is a fairly consistent one—higher biomarker levels tend to signal greater risk, even though a degree of variability and one contradictory finding remain.

## 4. Discussion

To the best of our knowledge, the present study is the first systematic review to investigate RC as a biomarker in predicting GDM. Currently, GDM is considered the most common medical complication during pregnancy. This disorder affects approximately 15% of pregnancies worldwide, accounting for nearly 18 million births annually [25]. The findings of the present systematic review, which included 11 high‐quality studies (with Newcastle–Ottawa scores of 8–10) from different countries, with a particular emphasis on Asian populations, highlight the potential role of RC as a promising candidate biomarker for the early detection of this important pregnancy complication.

Circulating RC, which consists of triglyceride‐rich lipoprotein remnants generated after the lipolysis of VLDL and chylomicrons, plays a key role in lipid metabolism and insulin resistance pathways [26]. Lipid metabolism undergoes substantial changes during pregnancy, and disturbances in this process are strongly associated with GDM and subsequent progression to Type 2 diabetes [27]. Recent studies have shown that increased insulin resistance leads to enhanced lipolysis, reduced lipoprotein lipase activity, and increased fatty acid biosynthesis, which together facilitate fat breakdown during the third trimester of pregnancy [28].

At the molecular level, the pathophysiology of GDM involves impaired secretion and signaling of incretin hormones, particularly reduced GLP‐1 and GIP, which compromise their insulinotropic effects [29]. In addition, proinflammatory cytokines such as TNF‐*α* and IL‐6 interfere with IRS‐1 phosphorylation and disrupt insulin‐dependent glucose uptake. Dysregulation of triglyceride transport and glucose uptake and thus the development of insulin resistance is primarily driven by impaired lipid metabolism, further exacerbated by oxidative stress and inflammation as secondary responses [28, 29].

The results of this systematic study indicate that the mean levels of RC in the GDM group (0.97) were significantly higher than those in the control group (0.71). This 36.6% difference in RC levels has considerable clinical significance and is consistent with the findings of Gao et al., who reported that RC levels during weeks 12–14 of pregnancy were positively associated with the risk of GDM, suggesting that RC may serve as an early warning indicator for GDM [23].

The quantitative association demonstrated in this study namely, a 9% increase in GDM risk with every 0.1‐unit increase in RC levels highlights the potential predictive power of this biomarker. This finding aligns with recent studies showing that elevated RC is independently associated with an increased risk of GDM and supports the recommendation that, in addition to traditional blood markers such as TG and TC, RC should also be considered as an additional biomarker for identifying women at risk of GDM [15]. Importantly, the potential value of RC should be considered within the broader framework of metabolic and inflammatory biomarkers rather than in isolation. Metabolic indices that reflect insulin resistance and dyslipidemia, such as the triglyceride–glucose (TyG) index and atherogenic index of plasma (AIP), may provide complementary information because they capture distinct aspects of metabolic dysfunction. A systematic review and meta‐analysis of five cohort studies involving 382,213 women reported that a higher first‐trimester or pre‐pregnancy TyG index was independently associated with subsequent GDM risk after adjustment for established confounders, including age, BMI, and family history of diabetes [30].

Recent evidence also suggests that metabolic biomarkers may retain greater independent predictive relevance than nonspecific inflammatory indices after adjustment for established metabolic and clinical risk factors. In a 2025 study of pregnancies complicated by pregestational diabetes, Yalcin et al. evaluated several first‐trimester inflammatory indices, including NLR, PLR, MLR, SII, SIRI, and CRP. Although several inflammatory indices were associated with adverse perinatal outcomes in univariate analyses, these associations did not remain independent after adjustment for maternal age, BMI, HbA1c, and parity; HbA1c and parity remained the significant independent predictors [31]. Similarly, a contemporaneous study evaluating first‐trimester AIP and TyG in women with pregestational diabetes found that TyG remained independently associated with several adverse perinatal outcomes after adjustment for age, BMI, parity, smoking, HbA1c, and fasting glucose, supporting the potential value of metabolic indices that capture insulin resistance beyond conventional glycemic measures [32].

These findings provide an important context for interpreting RC. Unlike inflammatory indices that may reflect a relatively nonspecific systemic inflammatory state, RC is directly related to the metabolism and clearance of triglyceride‐rich lipoproteins and may therefore more closely reflect the metabolic abnormalities underlying insulin resistance and dyslipidemia. Importantly, evidence specific to GDM supports this distinction. In a prospective cohort of 2528 pregnant women, the association between RC and GDM remained significant after mutual adjustment for TG, suggesting that the observed relationship was not simply attributable to elevated triglyceride concentrations [14]. More recently, a retrospective cohort of 1361 pregnant women reported that RC remained independently associated with GDM after adjustment for multiple covariates, including TG; notably, the association persisted in women with low TG but high RC, whereas the converse pattern was not observed to the same extent [15].

The relationship between metabolic and inflammatory pathways may nevertheless be interconnected rather than mutually exclusive. A prospective cohort study of 13,446 pregnant women reported a positive linear association between first‐trimester RC and subsequent GDM risk after adjustment for conventional lipid parameters and other potential confounders, whereas mediation analyses suggested that inflammation‐related factors may partially contribute to the association between RC and GDM [21]. Thus, RC may represent an integrated metabolic signal that is linked to both lipid dysregulation and low‐grade inflammation, whereas inflammatory indices alone may have limited specificity for the metabolic phenotype associated with GDM. However, direct head‐to‐head comparisons of RC with inflammatory and metabolic biomarkers within the same GDM populations remain limited. Consequently, the available evidence does not yet establish RC as superior to established metabolic biomarkers; rather, it supports further investigation of whether RC provides incremental predictive value when incorporated into multivariable models containing conventional metabolic and inflammatory markers.

The predominance of Asian studies in this systematic review particularly from China (seven studies) and South Korea (two studies) underscores the particular importance of this issue in this geographical region. A recent Korean study that examined the RC‐to‐HDL‐C ratio during the first trimester as a potential indicator for predicting GDM reflects a more advanced approach to the use of lipid ratios [22]. This geographical variation may be attributable to genetic differences, dietary patterns, and the higher prevalence of metabolic disorders in Asian populations.

The considerable variability in reported effect sizes across studies ranging from an OR of 1.05 in the study by Li et al. to 2.6 in the study by Shan et al. reflects the inherent complexities in the relationship between RC and GDM [9, 15]. This heterogeneity arises from the multifaceted interplay of factors, each of which may influence the strength and direction of the association. Methodologically, substantial differences in how RC is measured, the timing of blood sampling, and the diagnostic criteria for GDM represent major sources of variability. Some studies have employed the calculated formula RC = TC − LDL − C − HDL − C, whereas others have relied on direct measurement methods or alternative formulas, potentially leading to discrepancies in reported values [20, 24]. Furthermore, RC levels can be influenced by dietary fat intake, fasting status, and short‐term metabolic fluctuations, which may introduce additional variability in measurement and interpretation [33]. It is therefore essential to consider assay reproducibility when interpreting these findings, as methodological variability across laboratories and assay platforms may influence RC quantification and its comparability between studies. Future research should prioritize the standardization of RC measurement protocols to ensure analytical consistency and reliability across clinical settings.

In addition, demographic and clinical characteristics of the studied populations play a critical role in shaping this variability. Evidence indicates that pregnant women with elevated pre‐pregnancy BMI, high triglyceride levels, and elevated RC are at markedly increased risk of developing GDM [34]. Moreover, inconsistencies in controlling confounders across statistical models—such as physical activity, socioeconomic status, parity, and inflammatory markers—may have further influenced the reported associations.

Sample size is another key determinant of heterogeneity, with studies ranging from a few hundred to more than 33,000 participants. Smaller studies generally report wider CIs and have limited statistical power to detect modest associations, whereas large‐scale studies are better able to capture weaker but statistically significant associations and yield more robust results. Several studies have demonstrated that elevated RC levels at 12–14 weeks of gestation are positively associated with GDM risk, supporting the role of RC as an early warning marker for pregnant women [14, 21, 23].

A contradictory yet noteworthy finding from Sormunen‐Harju et al., which suggested a protective effect of RC in nonobese women, highlights the complexity of this relationship [20]. GDM in lean women has been recognized as a distinct entity that may require tailored management approaches, since insufficient insulin secretion rather than insulin resistance appears to be the dominant mechanism driving GDM in this subgroup. This finding raises the possibility of effect modification by BMI and underscores the importance of subgroup analyses. Epidemiological evidence further suggests that the association between GDM and dyslipidemia is mediated through maternal obesity, with higher BMI in early pregnancy linked to altered lipid metabolism, potentially contributing to increased GDM risk [35].

Recent metabolomics studies have shown that lean women with GDM remain at increased risk for subsequent diabetes and, despite a seemingly normal BMI, exhibit surprisingly high body fat percentages within 5 years postpartum [36]. These findings suggest that body composition and fat distribution may play a more critical role than overall BMI in modulating the effects of RC [37]. Furthermore, genetic differences across populations may also influence susceptibility to RC‐related diabetogenic effects, with certain ethnic groups demonstrating greater sensitivity [38].

Evidence also indicates that LDL cholesterol increases only in lean individuals with elevated BMI, which may help explain differential responses to RC across BMI categories [39]. Moreover, distinct pathophysiological mechanisms may underlie GDM in obese versus lean women: insulin resistance appears to predominate in obese women, whereas insulin deficiency is the primary driver in lean women [40, 41]. Such mechanistic differences may account for the divergent impact of RC observed across BMI groups.

Based on the findings of this study, RC shows promising potential for integration into routine GDM screening protocols. The advantages of using this biomarker include early detection (enabling the identification of high‐risk women during the first trimester), ease of measurement (obtainable through standard lipid tests), cost‐effectiveness (eliminating the need for complex and expensive assays), and combinability (the ability to be used alongside other biomarkers to enhance diagnostic accuracy). Nevertheless, it is important to acknowledge potential limitations of RC as a biomarker. These include variability in measurement techniques, potential overlap with triglyceride metabolism, and the influence of recent dietary intake or fasting duration on RC concentrations. Such factors may affect both the precision and clinical interpretation of RC levels.

However, before RC can be implemented as a standard clinical biomarker, further validation is required to confirm its analytical reproducibility across different assay platforms and populations. In addition, its clinical feasibility, particularly regarding cutoff determination, reference ranges, and integration with existing screening algorithms, should be thoroughly evaluated through large, prospective multicenter studies. Future studies should also directly compare the predictive performance of RC with established metabolic markers such as TG, TyG, AIP, and conventional glycemic measures, as well as inflammatory indices, using standardized models and clinically relevant performance metrics. Such head‐to‐head evaluations will be essential for determining whether RC offers incremental predictive value and whether its addition to existing metabolic risk models meaningfully improves early GDM risk stratification.

## 5. Conclusion

The findings of this systematic review indicate that circulating RC is a promising candidate biomarker for early risk stratification of GDM. The significant positive association between RC levels in the first trimester and the subsequent risk of GDM, together with biologically plausible mechanisms, highlights RC as a suitable candidate for integration into clinical protocols. However, it is important to acknowledge that predictive biomarkers such as RC should ideally be validated through large‐scale, multicenter, and ethnically diverse cohort studies to ensure their generalizability and robustness across different populations. Furthermore, future research should aimed at establishing standardized cutoff values, evaluating assay reproducibility, and assessing its clinical feasibility within real‐world healthcare settings to fully realize its diagnostic potential. Ultimately, RC may play a pivotal role in advancing early diagnosis and preventive management of GDM, thereby contributing to improved maternal and fetal outcomes.

## Author Contributions

**Fatemeh Abdi:** conceptualization, methodology, investigation, data curation, and writing—original draft**. Fatemeh Alsadat Rahnemaei:** investigation, literature screening, and data curation. **Fatemeh Khosravani:** data curation, formal analysis, quality assessment, writing—review and editing. **Farzad Sadri:** investigation and literature screening, data curation, quality assessment, writing—review and editing. **Yaser Mohammadi:** conceptualization, methodology, supervision, data curation, validation, writing—original draft, writing—review and editing, and project administration. All authors contributed to the interpretation of the findings and critically reviewed the manuscript.

## Funding

This study was supported by Iran University of Medical Sciences (10.13039/100012021; 34548).

## Disclosure

All authors have read and agreed to the final and published version of the manuscript. Yaser Mohammadi had full access to all of the data in this study and takes complete responsibility for the integrity of the data and the accuracy of the data analysis.

## Ethics Statement

This study was approved by the Ethics Committee of Iran University of Medical Sciences (Ethics Code: IR.IUMS.REC.1404.687).

## Consent

The authors have nothing to report.

## Conflicts of Interest

The authors declare no conflicts of interest.

## Supporting information


**Supporting Information** Additional supporting information can be found online in the Supporting Information section. Table S1: PRISMA 2020 checklist.

## Data Availability

The authors confirm that the data supporting the findings of this study are available within the article and/or its Supporting Information.
